# Testing and Evaluation of the Emulsifying Properties of Compound Oil Displacement Agents

**DOI:** 10.1021/acsomega.2c03653

**Published:** 2022-08-10

**Authors:** Leilei Zhang, Keliang Wang, Huiming An, Yu Su, Wei Zhang, Gen Li, Xinyi Yang

**Affiliations:** † Key Laboratory of Enhanced Oil and Gas Recovery Ministry of Education, Northeast Petroleum University, Daqing 163318, China; ‡ Baili College of Petroleum Engineering, Lanzhou City University, Lanzhou 730070, China

## Abstract

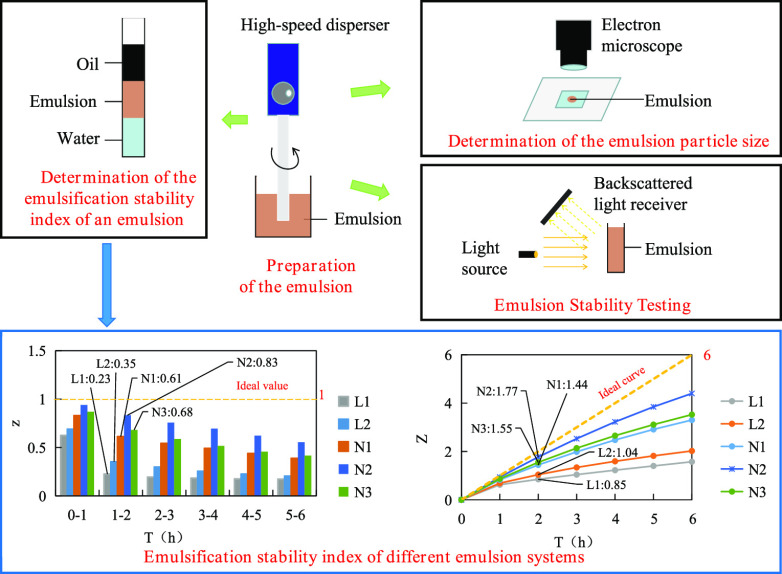

Aiming at the phenomenon that the emulsification degree of the composite oil displacement agents affects the recovery factor, composite oil displacement agents of the P/S binary system and the A/S/P ternary system were taken as research objects. Emulsion particle size and stability were tested and evaluated, and the effects of the surfactant and alkali content on the emulsification degree of emulsion were investigated. The concept of the emulsification stability index and its measuring method were put forward, and a method was used to test and evaluate the emulsification stability of the emulsion. The results showed that the emulsion formed by the ternary system had the smallest average particle size, the best stability, and the best emulsification stability. The binary composite system was second, and the polymer solution did not form an emulsion. The emulsification stability index method could effectively quantify the emulsification degree of the emulsion. Within a certain range, the increase of the surfactant and alkali content in the composite oil displacement agent was beneficial to the improvement of the emulsification degree of the emulsion.

## 1 Introduction

As an enhanced oil recovery technology, chemical flooding has been gradually extended to the development of various oil and gas fields, especially in China, where chemical flooding has become an important method for oil and gas field development to increase production and efficiency.^1−3^ In the process of chemical flooding development, compound oil displacement agents are showing their own advantages. Polymers, surfactants, and alkalis play a very important role in multi-component compound oil displacement agents and are widely used in various new oil displacement systems. The most notable feature of the polymer is that it can reduce the mobility of the displacement fluid, expand the swept volume, and reduce the remaining oil saturation.^4,5^ The most significant feature of the surfactant is that it can reduce the interfacial tension between oil and water so that crude oil can be easily peeled off from the rock surface and the residual oil saturation can be reduced.^6,7^ The most notable feature of the alkali is that it can react with oleic acid to form surfactants, which can react with other components in the oil displacement agent to improve oil displacement efficiency.^8,9^

In recent years, it has been found that under certain conditions, the emulsification between oil and water enhances oil recovery more than the effect of low interfacial tension. The effect of profile control and oil displacement produced by chemical oil displacement agents in contact, and emulsification with crude oil in reservoirs has attracted more and more scholars’ attention.^10−13^ During chemical flooding, surfactants are easily adsorbed on the surface of oil droplets to form stable emulsions. The emulsification between chemical flooding agents and crude oil and the properties of the formed emulsions have become research hotspots for oilfield development practitioners. In 1995, Aderangi and Wasam^14^ studied the coalescence of a single droplet at the liquid–liquid interface in a binary system oil–water emulsion composed of surfactants and polymers and found that interfacial tension is not strongly correlated with coalescence time or speed. In 2009, Angle^15^ studied the rheology of heavy crude oil emulsion and found that heavy crude oil emulsion can reduce the flow resistance of heavy crude oil. In 2013, Pei^16^ conducted an alkali flooding test for heavy oil reservoirs that were not suitable for thermal recovery. The results showed that the alkali solution penetrated into the crude oil and formed a W/O emulsion with the crude oil, which reduced the flow capacity of the water phase and increased the sweep efficiency. In 2015, Xuan^17^ studied the effect of polymers on emulsion stability in poly-surface binary composite flooding. The polymer was found to be beneficial to the stability of the emulsion. With the increase in shear rate, the stability became better and the droplet size became smaller. In 2017, Mandal and Chakraborty^18^ found in the experiments that the rheological trends of emulsions in a shear rheometer and porous media were consistent, and the rheological characteristics of emulsions were very different when the composition was different. In 2018, when Lu^19^ studied the rheological laws of emulsions in porous media, he found that the type, composition, dispersed phase concentration, and dispersed phase droplet size of the emulsions all affect it. The research on the emulsions formed by chemical oil displacement agents and crude oil mostly focuses on the rheological properties of emulsions and the effects of the viscosity of composite oil displacement agents and oil–water interfacial tension on the properties of emulsions. There are few systematic studies on the effect of the surfactant and alkali content in composite oil displacement agents on the emulsification degree of emulsion. Additionally, the evaluation method for the emulsification degree of emulsion needs to be improved.

This work systematically tested and evaluated the effects of alkalis and surfactants on the emulsification degree of emulsions. A new testing method for emulsification degree was proposed, and related concepts were introduced. The rationality of the method was demonstrated by comparing the test results of this method with the results of the classical emulsion particle size and Turbiscan Stability Index (TSI) test methods. These studies are important for emulsion control and flooding technology in oil and gas fields, as well as for determining the emulsification degree of emulsions.

## 2 Results and Discussion

### 2.1 Interfacial Tension

The interfacial tension of each scheme is shown in Table 1. It can be seen from Table 1 that the one-element system had no interfacial activity and had no ability to reduce the interfacial tension between the system and the simulated oil. The interfacial tension between the ternary system and the simulated oil reached an order of magnitude of 10^–3^, reaching the ultra-low interfacial tension standard. Changing the content of the surfactant and alkali could change the ability of the system to reduce the interfacial tension. The interfacial tensions of the binary system were 4.6 × 10^–2^ and 9.5 × 10^–3^ mN/m, respectively, both of which reached ultra-low interfacial tension but were higher than those of the ternary system, indicating that the interfacial tension between oil and the system could be reduced to a certain extent by only increasing the surfactant content when the components of the binary system remained unchanged.

**Table 1:** Interfacial Tension of Each Scheme

scheme	composition	interfacial tension/(10^–3^ mN·m^–1^)
1	HPAM polymer	
2a	surfactant/polymer (L1)	46.071
2b	surfactant/polymer (L2)	9.527
3a	alkali/surfactant/polymer (N1)	6.156
3b	alkali/surfactant/polymer (N2)	3.627
3c	alkali/surfactant/polymer (N3)	4.834

### 2.2 Average Particle Size of Emulsions in Different Systems

**Figure 1:**
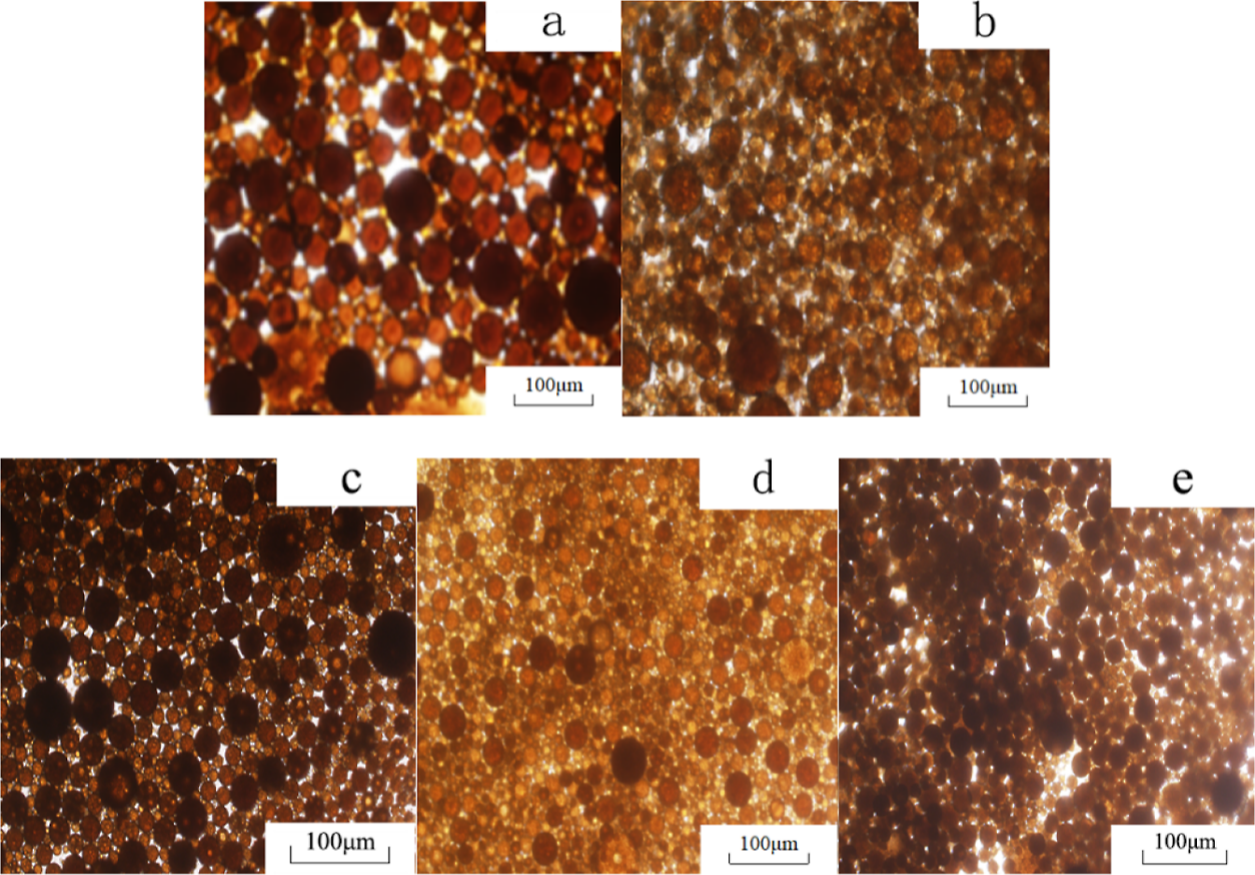
SEM images of emulsions. (a) L1; (b) L2; (c) N1; (d) N2; and (e) N3.

The interfacial tension between the oil displacement system and the crude oil is an important factor affecting the degree of emulsification. The emulsion droplets prepared by the above scheme systems and simulated oil were, respectively, placed under a microscope to observe the particle size distribution. The results are shown in Figure 1. The particle size of the emulsions is shown in Table 2.

**Table 2:** Particle Size of Emulsions

scheme	emulsion type	average particle size of emulsion/μm
1		
2a	O/W	45–50
2b	O/W	30–40
3a	O/W	15–30
3b	O/W	10–15
3c	O/W	15–20

It can be seen from Table 2 that the polymer solution did not have interfacial activity and did not form an emulsion. Both binary and ternary systems formed O/W emulsions, and the particle sizes of the emulsion in each system were different to some extent. The average particle size of emulsion droplets is related to the interfacial tension between the system and the simulated oil.^20,21^ After the surfactant is added to the system, the surfactant molecules are spontaneously adsorbed on the oil-system interface, which reduces the interfacial tension between the oil and the system, thereby reducing the interfacial energy, and the system gradually tends to a thermodynamically stable state. At the same time, surfactant molecules form an interfacial film with certain strength on the surface of oil droplets, making the structure of oil droplets more stable, which slows down the mutual attraction and coalescence of oil droplets.^22,23^ Therefore, the simulated oil temporarily exists in the form of small oil droplets, and the morphology of the emulsion observed under the microscope is shown in Figure 1a. With the increase in surfactant content, the interfacial tension is further reduced, the thermodynamic state of oil droplets is further stabilized, and the dispersion of oil droplets is enhanced. If the surfactant content continues to increase, the excess surfactant molecules in the continuous phase will entangle with each other to form micelles with a certain spatial structure. The micelles exist uniformly in the continuous phase, which sterically blocks the mutual attraction between oil droplets.^24,25^ Therefore, the average particle size of the emulsion droplets observed under the microscope becomes smaller, as shown in Figure 1b. For the ternary system, the addition of alkali will react with the acidic components in the crude oil to form active substances, which will have a synergistic effect with the original surfactant such that the interfacial tension between the oil and the system will be greatly reduced and the dispersion of oil droplets will be greatly increased.^26,27^ Therefore, the oil droplets appeared smaller under the microscope, as shown in Figure 1c–e.

### 2.3 Emulsion Stability of Different Systems

**Figure 2:**
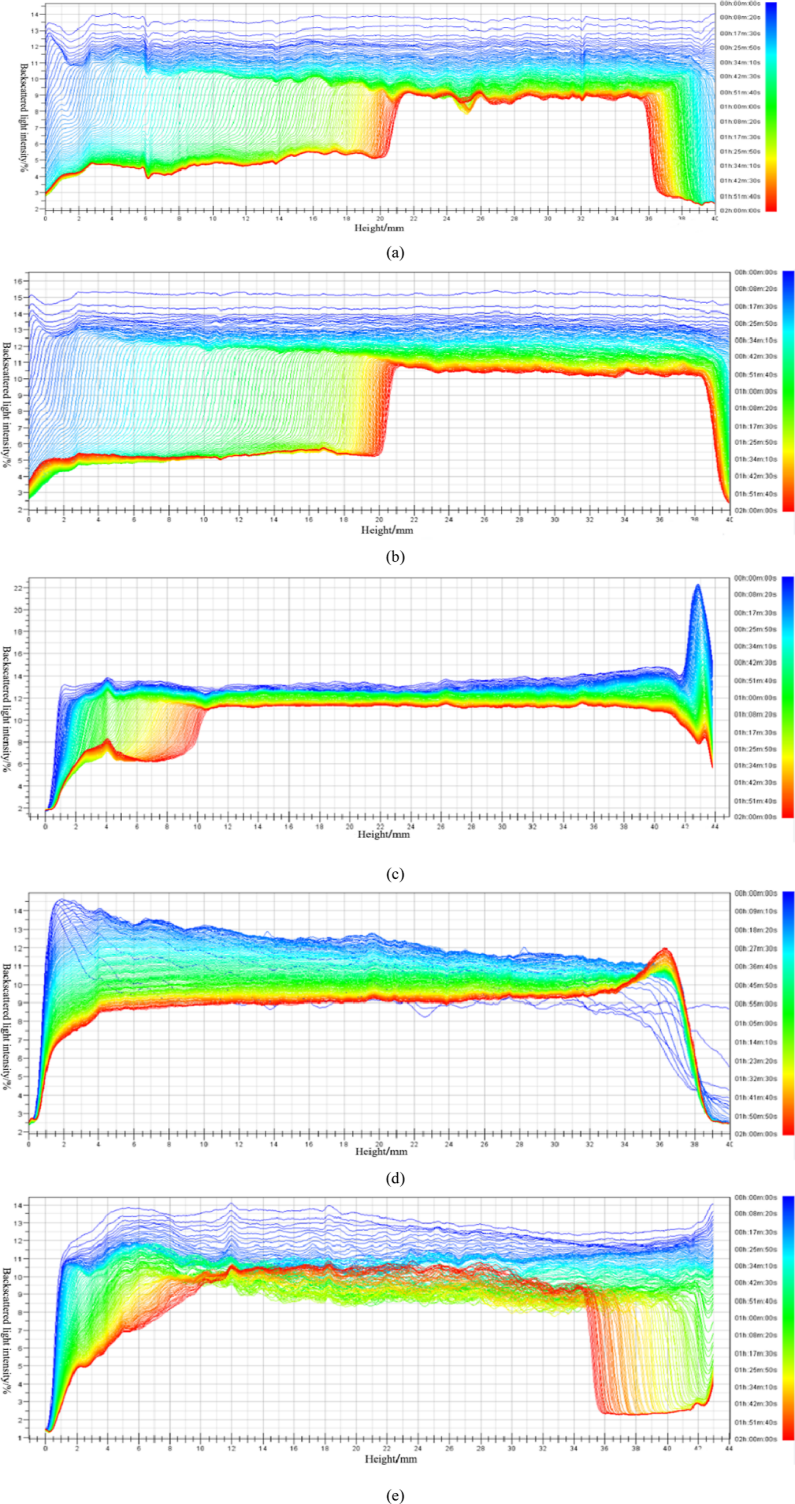
Variation of backscattered light intensity in different emulsions. (a) L1; (b) L2; (c) N1; (d) N2; and (e) N3.

The destabilization process of the emulsion within 2 h of each system was further studied. Figure 2 shows the changed curves of the backscattered light intensity of the emulsion system prepared by each system and the simulated oil. During the destabilization process of the emulsion, the emulsion droplets located in the lower part of the sample container float up. During the ascent, they aggregate with other droplets, coalesce into large droplets, and the large droplets continue to float and eventually merge into the upper oil phase, gradually realizing oil–water stratification.^28,29^ For the binary system, the whole sample container was all-emulsion at the beginning, the light transmittance was poor, and the backscattered light intensity was high. With the progress of the destabilization process, the intensity of the backscattered light in the lower part of the sample decreased greatly, and the light transmittance became better, indicating that the water content of the emulsion here increased, the emulsion droplets began to float, and the lower part gradually transformed into the water phase. After 2 h, the curves were divided into two obvious parts. The samples in the height range of 0–20 mm basically changed to the water phase, the samples in the height range of 20–21 mm were emulsions that were not completely broken, and the samples above a 21 mm height basically turned into the oil phase, as shown in Figure 2a,b. For the ternary system, the backscattered light intensity changed uniformly within 2 h, and the amplitude was small. At 2 h, there was no obvious difference in the backscattered light intensity between each part, and the transition between different phases was uniform, indicating that the emulsion was not obviously broken and had strong stability, as shown in Figure 2c–e. Therefore, the stability of the binary system emulsion was weaker than that of the ternary system emulsion.

**Figure 3:**
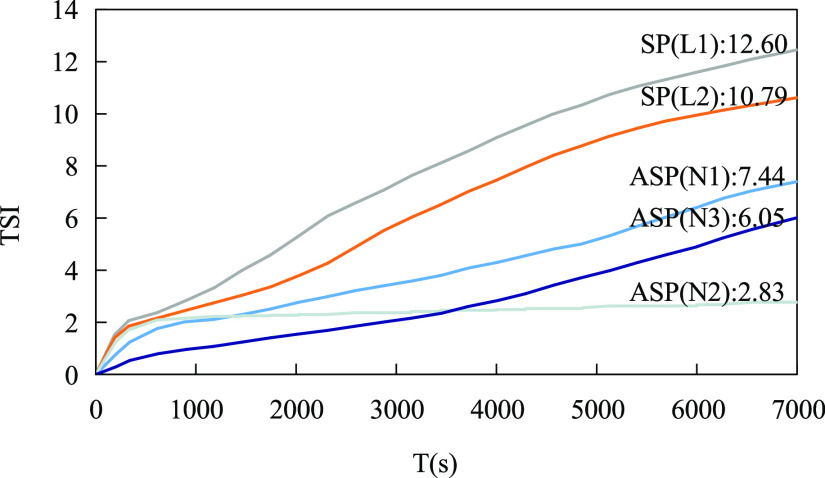
Changes in TSI values of various emulsions over time.

The TSI can quantitatively characterize the stability of the emulsion. The larger the TSI, the greater the fluctuation of the average backscattered light intensity in each scan, and thus the more unstable the system.^30,31^ The relationship between the TSI of the emulsion and the time is shown in Figure 3. The measurement time was 2 h, and the test temperature was 45 °C. It can be seen from Figure 3 that at the beginning of the test, there were three systems with similar TSI values, and the three TSI curves basically overlapped at the beginning. After 650 s, the TSI values of the alkali-free binary L1 and L2 systems began to increase. After 2 h, the TSI value of the alkali-free binary L1 system was 12.60, and the TSI value of the alkali-free binary L2 system was 10.79, showing only a difference of 1.81. It showed that under the condition that the system components remained unchanged, only increasing the surfactant content had little contribution to the stability of the emulsion. The TSI value of the ternary system N2 remained basically unchanged after 650 s, and the final TSI value was 2.83. The TSI values of the ternary systems N1 and N3 were higher than those of the N2 system, and they were 7.44 and 6.05, respectively, indicating that the ternary system N2 emulsion was the most stable, followed by N3 and N1, and both were stronger than the binary systems. At the same time, it showed that reducing the content of the surfactant and alkali would weaken the stability of the emulsion to varying degrees.

### 2.4 Emulsification Stability Index of Different Emulsion Systems

#### 2.4.1 Concept of the Emulsification Stability Index

**Figure 4:**
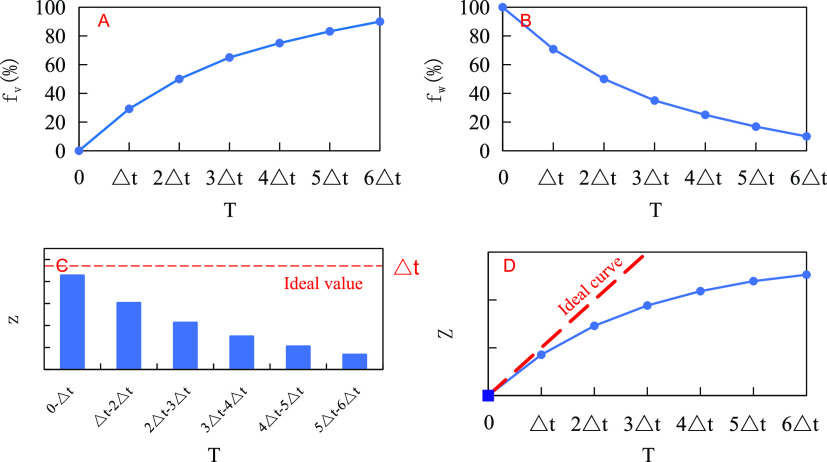
Schematic diagram of the concept of the emulsification stability index. (A) Schematic diagram of the water separation rate curve; (B) schematic diagram of the water retention rate curve; (C) schematic diagram of the step-length emulsification stability index; and (D) schematic diagram of the cumulative emulsification stability index.

In order to describe the emulsification strength and stability characteristics of the emulsion in the process of water shedding, three concepts, which are the emulsion water retention rate, step-length emulsification stability index, and cumulative emulsification stability index, were defined. (1) The water retention rate (fw): as shown in Figure 4B, in the process of water separation, fw is the ratio of the water content in the emulsion to the water consumption for preparing the emulsion. This value is calculated from the water separation rate (fv) of the emulsion, as shown in Figure 4A. (2) The step-length emulsification stability index: as shown in Figure 4C, the time of the water separation process is evenly divided into several sections; a single time span Δ*t* is the step size, and the area under the water retention rate curve within a single time span is used as the step-length emulsification stability index (*z*), which reflects the step-length emulsification stability of the emulsion during this period of time (the comprehensive performance of the emulsification strength and stability of the emulsion within this time range). The ideal step-length emulsification stability index value is 100% × Δ*t*. (3) The cumulative emulsification stability index: as shown in Figure 4D, the area under the water retention curve over a given time period is used as the cumulative emulsification stability index (*Z*), which reflects the emulsion’s cumulative emulsification stability over that time period, and the value is equal to the sum of all the step-length emulsification stability indexes over that time period. The slope of the ideal cumulative emulsification stability index curve is 1.

The water retention rate can indicate the emulsification strength of the emulsion at any time, and the higher the water retention rate at any time, the stronger the emulsification strength at that time. During the water separation process of the emulsion, the change in the water retention rate within a period of time represents the change in the emulsification strength of the emulsion within this period, and the change in the emulsification strength during this period reflects the stability of the emulsion within this period of time, and the greater the variation range of the emulsification strength, the worse the stability of the emulsion during this period. The step-length emulsion stability index reflects the comprehensive performance of the emulsification strength and stability of the emulsion in the long time range of any step. The smaller the step size, the more prominent the emulsification strength of the emulsion in the step size. When the step size is close to 0, it means that the emulsification strength of the emulsion at this moment is the water retention rate. The cumulative emulsification stability index reflects the comprehensive performance of the emulsification strength and stability of the emulsion during any period of time during the water separation process of the emulsion. When this time period is the time period of the whole emulsion water separation process, the cumulative emulsification stability index characterizes the comprehensive performance of the emulsification strength and stability of the emulsion during the whole time period.

#### 2.4.2 Calculation Method of the Emulsification Stability Index

The newly prepared emulsion was placed at a constant temperature, the volume of the water layer at different times was tracked and recorded, and the water separation rate *f*_v_, water retention rate *f*_w_, step-length emulsification stability index *z*, and cumulative emulsification stability index *Z* were calculated. The area under the water retention curve was calculated by the trapezoidal rule as the step-length emulsification stability index, and the cumulative summation of multiple step-length emulsification stability indexes was used as the cumulative emulsification stability index. They were calculated as follows.
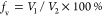
1
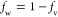
2
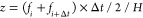
3
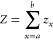
4where *f*_v_ is the water separation rate, *V*_1_ is the volume of the separated aqueous solution, mL, *V*_2_ is the volume of the oil-displacing agent solution used when preparing the emulsion, mL, *f*_w_ is the water retention rate, *z* is the step-length emulsification stability index, *i* is any time in the process of water separation of the emulsion, Δ*t* is the time step, h, *H* is the unit time, *H* = 1 h, *Z* is the cumulative emulsification stability index, and *z*_*x*_ is the emulsion stability index of any step length in the process of water separation of the emulsion, 0 ≤ *a* < *b*.

#### 2.4.3 Emulsification Stability of Different Emulsion Systems

**Figure 5:**
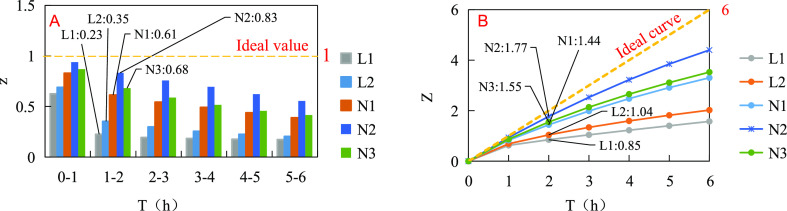
Emulsification stability indexes of different emulsion systems. (A) Step-length emulsification stability indexes. (B) Cumulative emulsification stability indexes.

The water separation process of the emulsion lasted for 6 h, and the step size Δ*t* = 1 h was set. The ideal step-length emulsification stability index was 100% × 1. The step-length emulsification stability index and the cumulative emulsification stability index curves of different emulsion systems are shown in Figure 5. The water separation phenomenon of the emulsions was mainly concentrated before 2 h. 0–1 h was the high-speed water separation period, 1–2 h was the differential water separation period of different systems, and after 2 h was the slow water separation period. The 1–2 h step emulsification stability index and the cumulative emulsification stability index at 2 h were selected as eigenvalues for analysis and description.

As shown in Figure 5A, the step-length emulsification stability indexes decreased with the prolongation of time, and the overall downward trend showed a rapid decline at first and then a slow decline. The better the emulsification stabilityis, the closer the step-length emulsification stability index is to the ideal step-length emulsification stability index value. The emulsification stability indexes of binary system emulsions decreased rapidly before 2 h. The 1–2 h step-long emulsification stability indexes were 0.23 and 0.35, respectively, which were much lower than 1, and after 2 h, step-length emulsification stability indexes were always at a low value. These indicate that before 2 h, the binary system emulsions have fast water separation, low water retention rate, and poor emulsification stability. After 2 h, the step-length emulsification stability is always poor. In contrast, the emulsification stability indexes of the ternary system emulsions decreased slowly before 2 h. The emulsification stability indexes of the 1–2 h step were 0.61, 0.83, and 0.68, respectively, which were slightly less than 1, and after 2 h, the step-length emulsification stability indexes were still at a high value. These indicate that before 2 h, the water separation rate of the ternary system emulsions is slower, the water retention rate is higher, and the emulsification stability is better. The step-length emulsification stability remains better after 2 h.

As shown in Figure 5B, the cumulative emulsification stability indexes increased with time, and the overall upward trend showed a rapid rise at first and then a slow rise. The cumulative emulsification stability index at any time point reflects the comprehensive emulsification stability of the emulsion before that time point. The better the emulsification stability, the closer the cumulative emulsification stability index is to the ideal cumulative emulsion stability index curve. The cumulative emulsification stability indexes of the binary system emulsions increased rapidly before 2 h. The cumulative emulsification stability indexes at 2 h were 0.84 and 1.04, respectively, which were far from the ideal curve. After 2 h, the cumulative emulsification stability indexes increased slowly and remained at a low value. These indicate that the binary system emulsions have a fast water separation rate, low water retention rate, and poor cumulative emulsification stability between 0 and any time. In contrast, the cumulative emulsification stability indexes of the ternary system emulsions before 2 h increased faster. The cumulative emulsification stability indexes at 2 h were 1.44, 1.77, and 1.55, respectively, which were closer to the values of the ideal curve. After 2 h, the cumulative emulsification stability indexes still maintained a rapid growth trend. These indicate that the ternary system emulsions have a slow water separation rate, a high water retention rate, and good cumulative emulsification stability from 0 to any time.

**Figure 6:**
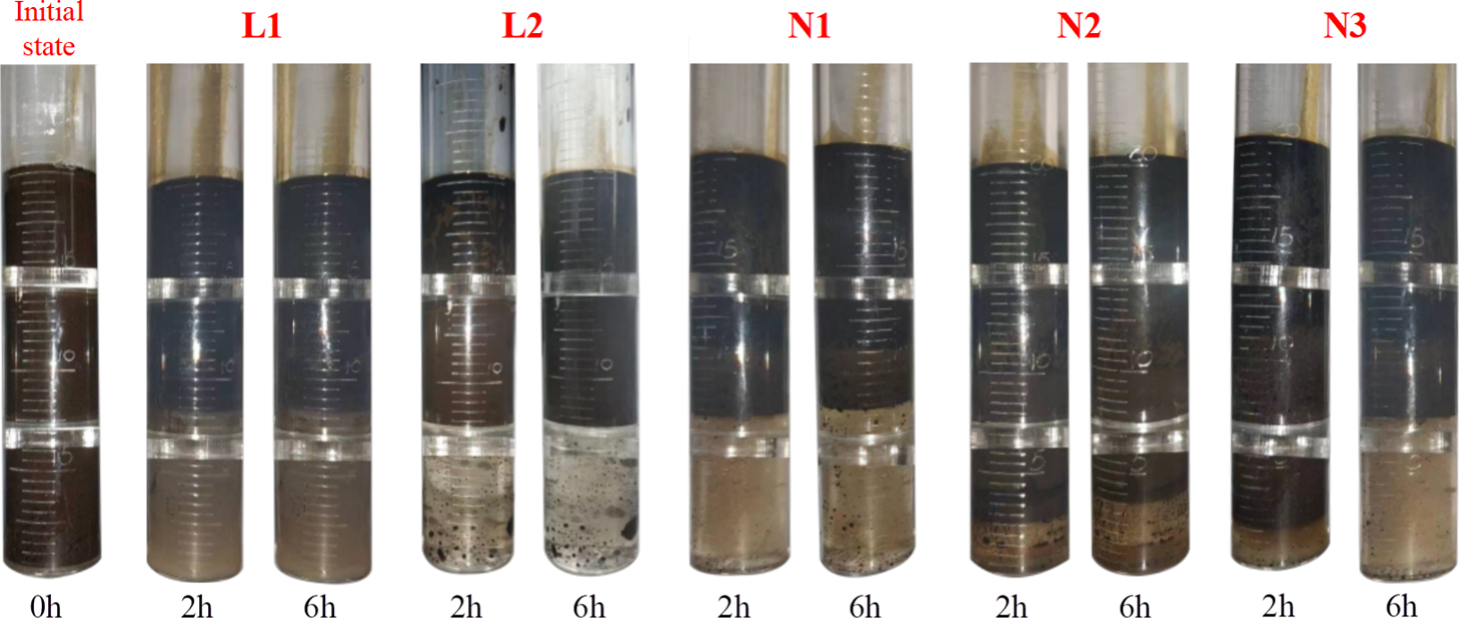
State of emulsions at different water separation times.

The relationships between the eigenvalues of the step-length emulsification stability indexes and the cumulative emulsification stability indexes of the five composite oil displacement agent emulsions were N2 > N3 > N1 > L2 > L1. Therefore, their emulsification stability from high to low was N2 > N3 > N1 > L2 > L1. Figure 6 shows the initial state, the state at 2 h, and the state at 6 h of the five-scheme emulsions.

#### 2.4.4 Rationality of the Emulsification Stability Index Method

The smaller the particle size of the dispersed phase of the emulsion, the stronger the emulsification degree. The smaller the TSI value of the emulsion, the stronger the emulsification degree. The larger the emulsification stability index, the stronger the emulsification degree. The characterization values of the emulsification degree of the emulsions formed by the five kinds of composite oil displacement agents in the three abovementioned methods were compared, as shown in Table 3 below.

**Table 3:** Emulsification Degree Characterization Values

scheme	average particle size of emulsion/μm	TSI value	*z* (1–2 h)	*Z* (2 h)
L1	45–50	12.6	0.23	0.85
L2	30–40	10.79	0.35	1.04
N1	15–30	7.44	0.61	1.44
N2	10–15	2.83	0.83	1.77
N3	15–20	6.05	0.68	1.55
size relationship	N2 < N3 < N1 < L2 < L1	N2 < N3 < N1 < L2 < L1	N2 > N3 > N1 > L2 > L1	N2 > N3 > N1 > L2 > L1
emulsification degree	N2 > N3 > N1 > L2 > L1			

As can be seen from Table 3, the three methods had the same test results for the emulsification degree of the five kinds of emulsions. The emulsification degree of the emulsions formed by the ternary system was higher than that of the binary system. Within a certain range, the increase of the surfactant and alkali content in the composite oil displacement agent was beneficial to the improvement of the emulsification degree of the emulsion. In the emulsification stability index method, the selection of the step size, the preparation method of the emulsion, the test environment, and the characteristic value need to be further studied and determined in order to form the emulsification stability test standard of the emulsion.

## 3 Conclusions

(1) The lower the interfacial tension between the oil and the system, the better the thermodynamic stability of the emulsion and the smaller the average particle size of the dispersed phase. The relationship between the emulsification degrees of the composite system emulsion is ternary system > binary system, and the polymer solution did not form an emulsion.
(2) The instability rate of the binary system emulsions was fast, and the stability of the emulsions was poor. The active substances generated by alkali and oleic acid had a synergistic effect with the surfactant in the ternary system, which greatly increased the stability of the emulsions.
(3) The concept and algorithm of the emulsification stability index were proposed, and the method of the emulsification stability index could effectively quantify the emulsification stability of the emulsions.
(4) Within a certain range, the increase of the surfactant and alkali content in the composite oil displacement agent was beneficial to the improvement of the emulsification degree of the emulsions.

## 4 Experiments

### 4.1 Experimental Materials and Instruments

Petroleum sulfonate [effective content of 40% (w)] and partially hydrolyzed polyacrylamide (HPAM) [relative molecular weight 12 to 16 × 10^6^ and solid content 90.6% (w)] were purchased from the PetroChina Daqing Refining and Chemical Company. Sodium carbonate, analytically pure, was obtained from the Tianjin Kaitong Chemical Reagent Co., Ltd. CNPC Daqing Oilfield B oil production plant combined station exports sewage, which was used as experimental water. The water quality analysis is shown in Table 4.

**Table 4:** Analysis of Water Quality of Exported Sewage

	Na^+^ (mg/L)	K^+^ (mg/L)	Ca^2+^ (mg/L)	Mg^2+^ (mg/L)	HCO_3_^–^ (mg/L)	CO_3_^2–^ (mg/L)	Cl^–^ (mg/L)	total salinity (mg/L)
salinity	2669.3	21.5	14.1	2.4	1026.8	141.1	1547.2	5458.1

The IKA T25 high-speed disperser is manufactured by the German IKA company. The Model 500 spinning drop interfacial tensiometer is obtained from the Texas Corporation, USA. The Brookfield DV-II viscometer is purchased from the Brookfield Company in the United States. The Olympus IX73 electron microscope is received from the Japanese Olympus Company. The Turbiscan Lab stability analyzer is purchased from the French Formulaction Company.

### 4.2 Preparation of Simulated Oil

The simulated oil used in the experiment was a mixture of crude oil, in the combined station of the B oil production plant of Daqing Oilfield, and kerosene in a volume ratio of 5:1. At 45 °C, and it had a viscosity of 10 mPa·s. The purpose of using the simulated oil was to make its viscosity consistent with the formation conditions. Although the change in the oil phase composition will affect the emulsifying ability and reduce the oil–water interfacial tension to a certain extent, the effect of this change is much smaller than that of adding a surfactant and an alkali to the water phase. Besides, the same simulated oil was used in each experiment, so the influence of kerosene on the experimental results can be ignored.

### 4.3 Determination of Interfacial Tension

The interfacial tension was measured by the spinning drop method. In the experiments, the temperature was set to 45 °C, and the rotational speed was set to 5000 rpm. After 2 h of starting the test, equilibrium was reached, and the interfacial tension no longer changed and was recorded this time.

### 4.4 Determination of the Emulsification Degree

**Figure 7:**
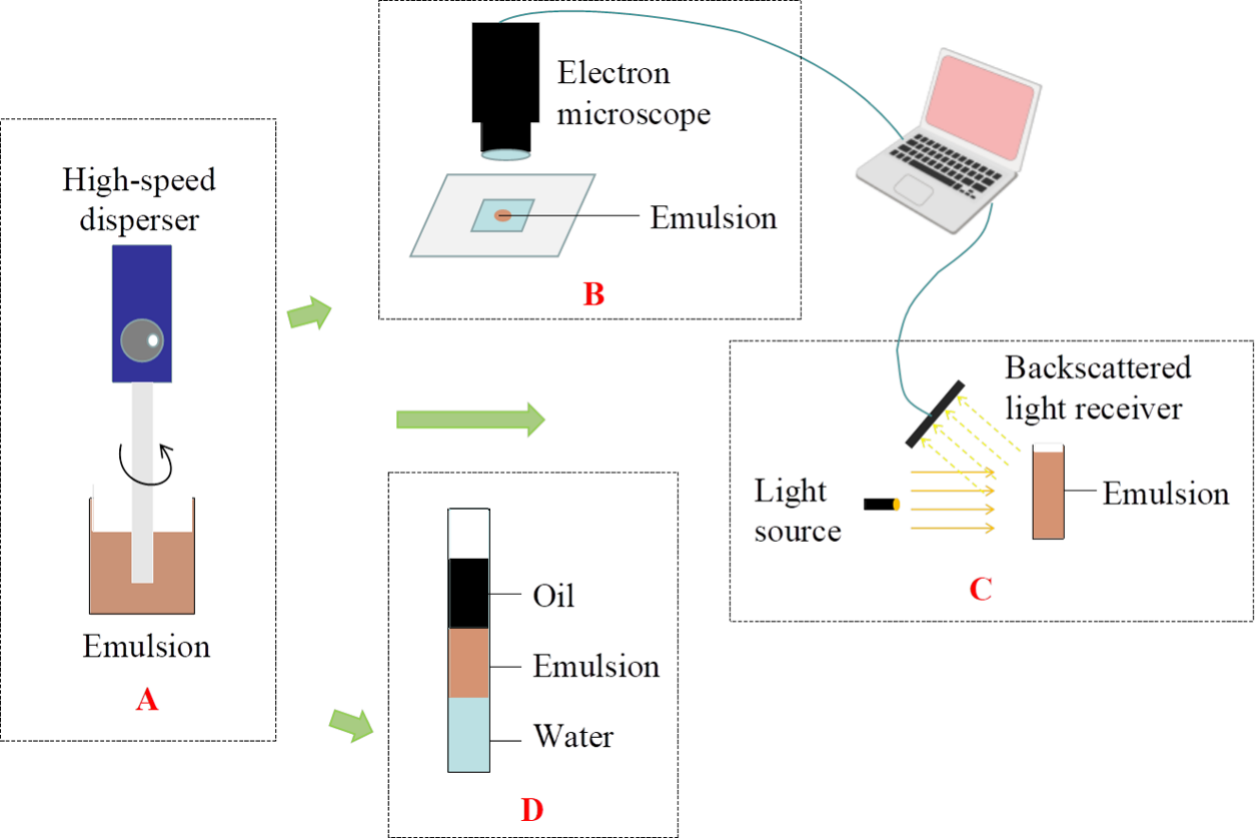
Emulsion preparation and emulsification degree test schematic diagram. (A) Preparation of the emulsion; (B) determination of the emulsion particle size; (C) emulsion stability testing; and (D) determination of the emulsification stability index of an emulsion.

Preparation of the emulsion: 50 mL of oil-displacing agent and simulated oil were taken in a beaker at a volume ratio of 1:1, preheated in a water bath at 45 °C for 10 min, and stirred at 4000 rpm for 5 min to obtain the emulsion shown in Figure 7A.

Determination of the particle size of the emulsion: one drop of the emulsion on a glass slide was taken, the type of the emulsion was observed through an electron microscope, the uniformity of the distribution of the emulsion droplets was recorded, and the average particle size of the emulsion droplets was measured, as shown in Figure 7B.

Emulsion stability testing: the emulsion was taken into a sample bottle and analyzed with a stability analyzer, resulting in an output of the backscattered light intensity curve and TSI, as shown in Figure 7C.

Determination of the emulsification stability index of the emulsion: 20 mL of emulsion was taken into a glass-graduated cylinder, the water separation rate was recorded every 1 h, and the step-length emulsification stability index *z* and cumulative emulsification stability index *Z* from the water separation rate were recorded, as shown in Figure 7D. Refer to “Emulsification Stability Index of Different Emulsion Systems” for the parameter concepts and calculation methods involved.

### 4.5 Design of Experimental Schemes

Scheme 1 was a one-component system composed of HPAM solution, which was used for comparison with composite oil displacement agents.

Scheme 2 was a binary system. The emulsifying ability of the system was changed by changing the surfactant content. Therefore, two schemes with different surfactant contents were designed: Scheme 2a is a binary system L1 composed of HPAM solution and 0.15% (w) petroleum sulfonate solution, and Scheme 2b was a binary system L2 composed of HPAM solution and 0.30% (w) petroleum sulfonate solution.

Scheme 3 was a ternary system. The emulsifying ability of the system was changed by changing the content of the surfactant and alkali. Therefore, three schemes with different contents of surfactant and alkali were designed, respectively: Scheme 3a was a ternary system N1 composed of HPAM solution, 0.15% (w) petroleum sulfonate solution, and 1.2% (w) sodium carbonate solution. Scheme 3b was a ternary system N2 composed of HPAM solution, 0.30% (w) petroleum sulfonate solution, and 1.20% (w) sodium carbonate solution. Scheme 3c was a ternary system N3 composed of HPAM solution, 0.30% (w) petroleum sulfonate solution, and 0.60% (w) sodium carbonate solution.

In the above schemes, the viscosity of the systems was controlled at 40 mPa·s (a temperature of 45 °C and a shear rate of 6.0 rpm) by adjusting the content of HPAM.
